# Incidence and predictors of initial antiretroviral therapy regimen change among children in public health facilities of Bahir Dar City, Northwest Ethiopia, 2021: multicenter retrospective follow-up study

**DOI:** 10.1186/s12887-022-03256-8

**Published:** 2022-04-08

**Authors:** Molla Azmeraw, Yinager Workineh, Friehiwot Girma, Amare Kassaw, Gashaw Kerebeh, Abraham Tsedalu, Agimasie Tigabu, Teshale Mengesha, Eleni Dagnaw, Dessie Temesgen, Biruk Beletew, Getenet Dessie, Melsew Dagne

**Affiliations:** 1 Department of Nursing, College of Health Sciences, Woldia University, P. O. Box: 400, Woldia, Ethiopia; 2 Department of Pediatrics and Child Health Nursing, College of Medicine and Health Science, Bahir Dar University, Bahir Dar, Ethiopia; 3 Department of Pediatrics and Child Health Nursing, College of Medicine and Health Science, Debre Tabor University, Debre Tabor, Ethiopia; 4 Department of adult Health Nursing, College of Medicine and Health Science, Debre Tabor University, Debre Tabor, Ethiopia; 5 Department of Pediatrics and Child Health Nursing, College of Health Science, Dire Diwa University, Dire Diwa, Ethiopia; 6 Department of Pediatrics and Child Health Nursing, College of Medicine and Health Science, Debre Birhan University, Debre Birhan, Ethiopia; 7 Department of Adult health Nursing, College of Medicine and Health Science, Bahir Dar University, Bahir Dar, Ethiopia

**Keywords:** Children, Human immunodeficiency virus, Antiretroviral therapy, Initial regimen change, Ethiopia

## Abstract

**Background:**

The inconsistent use of antiretroviral therapy can lead to the risk of cross-resistance between drugs. This reduces subsequent antiretroviral drug options. The burden of initial antiretroviral therapy ranges from 11.3% in South Africa to 71.8% in Malaysia. There is evidence that it is important to maintain children’s initial antiretroviral therapy regimens. However, the incidence and predictive factors of initial antiretroviral therapy regimen changes in the research context are still unknown in the study setting. **So,** the study was aimed to assess incidence and predictors of initial antiretroviral therapy regimen changes among children in public health facilities of Bahir Dar city.

**Methods:**

A retrospective follow-up study was conducted in 485 children who received antiretroviral therapy between January 1, 2011 and December 30, 2020. These children were selected using simple random sampling techniques. The data were entered by Epi data 3.1 and the analysis was completed by STATA 14.0. The missing data was treated with multiple imputation method. The data were also summarized by median or mean, interquartile range or standard deviation, proportion and frequency. The survival time was determined using the Kaplan Meier curve. The Cox Proportional Hazard model was fitted to identify predictors of initial antiretroviral therapy regimen change. The global and Shoenfeld graphical proportional hazard tests were checked. Any statistical test was considered significant at *P*-value < 0.05. Finally, the data were presented in the form of tables, graphics and text.

**Result:**

Among the 459 study participants, 315 of them underwent initial regimen changes during the study accumulation period. The shortest and longest follow up time of the study were 1 month and 118 months, respectively. The overall incidence rate of initial regimen change was 1.85, 95% CI (1.66–2.07) per 100 person-month observation and the median follow up time of 49 (IQR 45, 53) months. The independent predictors of initial regimen changes were poor adherence (AHR = 1.49, 95%CI [1.16, 1.92]), NVP based regimen (AHR = 1.45, 95%CI [1.15, 1.84]) comparing to EFV based regimen, LPVr based regimen (AHR = 0.22, 95%CI: (0.07, 0.70)) comparing to EFV based regimen, history of tuberculosis (AHR = 1.59, 95%CI [1.14, 2.23]) and being male (AHR = 1.28, 95%CI [1.02, 1.60]).

**Conclusions and recommendations:**

In this study, the incidence of initial regimen change was high. The risk of initial regimen change would be increased by being male, poor adherence, having history of tuberculosis and NVP based initial regimen. Therefore, strengthening the health care providers’ adherence counseling capability, strengthening tuberculosis screening and prevention strategies and care of initial regimen type choice needs attention in the HIV/AIDS care and treatment programs.

## Background

Although various plans and policies have been implemented in the past 40 years to combat the impact of HIV/AIDS, the current reduction in HIV/AIDS-related morbidity and mortality is also rested on early diagnosis, early initiation and continuous use of antiretroviral therapy. The inconsistent ART use increases the likelihoods of adverse outcomes in HIV care and treatment programs, including treatment failure [1, 2].

Antiretroviral therapy regimen change (ARTRC) refers to the substitution of one or more of the antiretroviral drugs in the initial antiretroviral therapy (ART) regimen. The drugs may belong to the same or different drug classes [3, 4]. If the antiretroviral treatment regimen is changed for the first time, it is called an initial regimen change (IRC). Often, changes in the antiretroviral treatment regimen will occur as long as the child receives HIV treatment and care for a variety of reasons within a few months and beyond. The reasons may be market availability, toxicity, treatment failure, drug stock-out, or simplification [5]. A one or more ART drugs can also be changed from the initial regimen to another regimen, depending on the patient’s condition and recommendations from WHO or specific country guidelines [6]. Before making changes to the patient’s ART regimen, clinicians will consider the patient’s past regimen, past ART failure episodes, past drug resistance test results, drug costs, and the patient’s ability to tolerate the new regimen [7].

The magnitudes of RC varied across the previously conducted studies in children [3, 8, 9]. In America and Malaysia, the prevalence of initial regimen change among children was 72% [8] and 71.8% [3], respectively. Moreover, a randomized trial of PIs and NNRTIs in the United States of America showed that 72% of participants experienced at least one treatment disruption during 8 years of follow up [8]. In Spain, a follow-up study among 212 HIV-infected children also reported that only 24% of children remained on their first combination therapy [10]. In addition, a prospective study in Ghana reported that 3.3% of the participants had experienced initial RC [11]. Another study in South Africa also revealed that overall incidence of initial ARTRC was 85.9 per 1000 patient-years, only 72 and 26% of children were remained on initial regimen at three and 5 years of ART during 10 years of study follow-up, respectively [12]. Another two different studies in South Africa showed that 11.3 and 26% of the study participants experienced IRC [12, 13]. Similarly, 2 years regimen durability study in South Africa revealed that there were about 15.3% single-drug substitutions, 7.2% treatment interruptions and 3.3% switched to second-line therapy [9].

The initial or subsequent RC could be affected by a variety of factors which might vary greatly with baseline laboratory values CD4 count [14], co morbidity [8, 15], initial regimen type [8, 14], having poor adherence [8, 12], the occurrence of adverse drug reaction/toxicity (ADR) [14], treatment failure [8, 12, 14] and availability of new drug [15]. These factors often cause significant morbidity and antiretroviral treatment failure. The predisposing factors were also varying across the findings of the previous studies [3, 12, 14, 16].

A serious confront of ART drug substitution, switching, interruption and discontinuation are the emergences of drug resistance, making future therapeutic interventions ineffective and narrowing the subsequent possible alternatives [17]. Additionally, RC also affects the success of HIV treatment to achieve Joint United Nation Program on HIV/AIDS (UNAIDS) 90–90-90 and 95–95-95 goals [18, 19].

In order to improve success of the initial ART regimen in HIV care and treatment program; optimizing the limited available ART regimens is vital [20]. Several studies done on predictors of first line HIV treatment failure were also reported that ART regimen change is a predictor of the subsequent ART failure [21, 22]. Even though the availability of new drugs has improved the patient acceptance, satisfaction to treatment, and safety profile of new regimens, the definition of the best available starting ART regimen is hindered by the lack of data on the exact impact of long-term durability of initial ART regimens [23]. As a result, the knowledge of how often a combination regimen is changed can be a good substitute marker of the regimen success. Even though several studies have tried to determine incidence and predictors of initial ARTRC, they were not consistent. So far, there were a limited number of studies on predictors of initial ARTRC among children in Ethiopia.

Therefore, this study aimed to determine incidence and predictors of initial ARTRC among children in public health facilities of Bahir Dar city, Northwest Ethiopia, 2021.

## Methods

### Study design, period and setting

Institution based retrospective follow-up study was conducted in Bahir Dar city from January 1, 2011 to December 31, 2020. Bahir Dar is the capital city of Amhara regional state located in Northwest Ethiopia and 565 km far from Addis Ababa, the capital city of Ethiopia. It also covers an area of 28km^2^ and situated 1800 m above sea level. The current projected population estimation is 389, 177. Of these 147,983 are children under the age of 15 years. The city administration has three public hospitals and 10 health centers. Pediatrics antiretroviral therapy services have been delivered in all public health facilities except Zegie health center in the city. Zegie health center have been provided only prevention of mother to child HIV transmission treatment service. Furthermore, Tibebe Ghion Specialized Comprehensive Hospital and Meshenti health center have no cases on ART under the age of 15 years. So, the study population was from the rest 10 health facilities. In the last 10 years, from January 1, 2011 to December 31, 2020, 747 HIV positive children had been enrolled in ART centers of the study area.

### Population

All HIV positive children aged below 15 who were enrolled in all public health facilities of HIV care and treatment centers in Bahir Dar City from January 1, 2011 to December 31, 2020 were the study population. All HIV positive children aged below 15 who started ART in all public health facilities of HIV care and treatment centers in Bahir Dar City from January 1, 2011 to December 31, 2020, were included where as all participants who were transferred in and who had only one visit were excluded.

### Variables

Time to initial ART regimen change was the dependent variable. The independent variables were age, sex, parental aliveness, primary care giver HIV status, primary care giver, primary HIV confirming service area, catchment area, base line CD4 count, base line hemoglobin, base line nutritional status, base line functional status, base line opportunistic infection, base line weight, previous exposure for ART drugs, initial regimen, drug class, history of poor adherence, history of tuberculosis, WHO clinical stage deterioration history.

### Sample size determination

The sample size was determined using the double population proportion difference formula using Epi Info version 7 software by considering the major predictor variables were regimen type, WHO immunological status and adherence from previously conducted studies in South Africa, Malaysia Europe and Thailand [9, 14, 15]. From those predictor variables, the sample size that we obtained from WHO immunological status had given the final sample size of the study. It gave maximum sample size when compared to other predictors. A proportion of exposed (23%) and non-exposed (16%), one to one exposed to non-exposed ratio (1:1), 95% level of confidence interval, 1.26 hazard ratio and power of 80% was yielded the largest estimated sample size, 1060 individual charts. Then, the largest calculated sample size, 1060 was larger than the reference population. So, correction formula was required.

Let, n_i_ = the calculated sample size, *N* = the population, and *n* = the final required sample size.

*n* $=\frac{\mathrm{ni}}{1+\mathrm{ni}/\mathrm{N}}$, $\frac{1060}{1+1060/747}\sim442$, then 10% contingency for incomplete charts was considered.

Finally, the minimum required sample size was 485.

### Sampling technique and sampling procedure

**Fig. 1:**
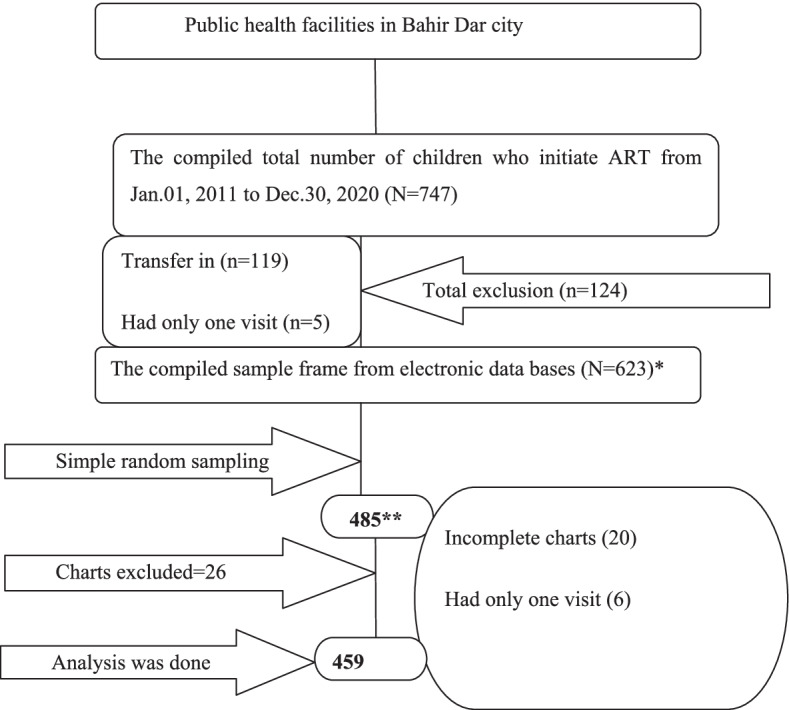
Schematic presentation of sampling procedure to assess initial ART regimen change among children in public health facilities of Bahir Dar city, Northwest Ethiopia, 2021. * Bahirdar HC =98, shumabo HC = 1, Tisabay HC =2, Han HC =77, Abay HC =63, Shimbit HC =12, Adis alem primary hospital = 9, Felege hiwot specialized comprehensive hospital = 361. ** Bahirdar HC =76, shumabo HC = 1, Tisabay HC =0, Han HC =66, Abay HC =51, Shimbit HC =10, Adis alem primary hospital = 8, Felege hiwot specialized comprehensive hospital = 273 (NB:HC=Health center)

All public hospitals and health centers in Bahir Dar city that has provided ART services were included in the study. Initially, list of the medical record numbers of the children in each ART clinics was prepared. Then, the lists of medical record numbers from all ART clinics were combined to make a single sample frame from the electronic data base (smart care) of each facility. Finally, the study participants were selected through computer generated random number (Fig. 1).

### Data collection tool and procedures

In order to collect the data from charts, the structured questionnaire was prepared. The questionnaire was adapted from the Ethiopian Federal Ministry of Health HIVAIDS care and treatment follow-up forms. The laboratory requests and history sheet documentation were also considered in preparing the questionnaire. Then, data were collected from both patient charts and electronic data base in terms of socio-demographic variables, base line Clinical and laboratory related factors and treatment-related factors of HIV infected children. Charts were accessed based on their medical record numbers. One data clerk personnel for 1 day and two card room workers during the data collection period were recruited. The data were collected by three BSc nurses who are working at HIV care and treatment clinics from Han health center, Abay health center and Adis alem primary hospital. The supervision was done by both supervisor and principal investigator.

### Data quality assurance

Before the data collection, a pretest was done among 25 medical records of children enrolled on ART from year January 1, 2011-December 30, 2020 at Han health center and Felege hiwot comprehensive specialized hospital. Data quality was also guaranteed with a proper recruitment and training of data collectors. The two data collectors have basic comprehensive HIV care and treatment training and the rest one data collector has also ART mentoring certificate. Two supervisors closely supervised the data collection process every day. At the end of each data collection date, the data completeness and consistency was cross-checked by supervisor and principal investigator. In addition, once the data were extracted from patient charts, the charts were coded to avoid duplication.

### Data processing and analysis

After the data extracted from the charts, it were checked for consistency and completeness. Then, data were coded and entered into Epi Data version 3.1. Next, they were exported to STATA version 14.0 and cleaned. The mean with standard deviation in the case of normally distributed variables, otherwise median with inter-quartile range (IQR) were used as descriptive and summary statistics for continuous variables. Frequencies and proportions were also used to describe the characteristics of categorical variables. The missing data was handled via multiple imputations for CD4 count and hemoglobin level. Kaplan–Meier survival curve was used to estimate median time. The incidence of initial ART regimen change during the follow up period was calculated and presented as per 100 person month’s observation. Cox Proportional Hazards regression model fitness was checked by using Shoenfeld residuals test (global test) together with graphical test. The assumptions were met for both tests. In addition, the overall goodness of model fitness was checked via Cox Snell residual. The Nelson Aalen hazard function follows the 45^0^ line very closely except for very large values of time. So, it can be concluded that the final model fits very well.

Then, the bivariable analysis was done to identify associations between dependent and each independent variable.

Variables with *P* ≤ 0.25 levels in the bivariable analysis were considered as candidate variable to be entered into the multivariable analysis. The multi co linearity of the candidate variables was checked using Cramer’s V correlation coefficient. Adjusted hazard ratio, 95% CI and *P*-value was used to assess the strength of association and statistical significance. Any statistical test was considered significant at *P*-value < 0.05.

### Operational definitions

In this study, event was occurrence of initial ART regimen change. Initial ART regimen change defined as either first-time substitution of at least one drug from the original regimen within or out of the drug class including complete switch of the entire regimen. Time-to-event (ART regimen change) was calculated by subtracting the date of antiretroviral therapy (ART) regimen initiation from the date of the event occurred or censored. Survival status was the outcome of children; either ART regimen changed or censored. Patients with the first date of lost to follow up, defaulters, transfer out, death before the end of the follow-up period and completed the follow-up period without developing the event was considered as censored. Date of ART initiation (month “0”) and date of initial regimen change or censoring was used as starting and end time of study follow up, respectively. In addition, month was used as measurement scale. Regarding to chart incompleteness, the chart is said to be incomplete when there was unknown outcome status and/or unknown ART initiation date.

## Result

### Socio-demographic related characteristics

Among 485 participants, 459 were included for analysis. About 315 (68.63%) of them undergone first-line antiretroviral therapy regimen changes. Of which 91.11% (*n* = 287) were undergone drug substitution and the rest 28(8.89%), 95%CI (27.28, 35.78) were switched to second-line ART regimen. The socio-demographic characteristics of the participants were summarized below (Table 1).

**Table 1:** Base line socio demographic characteristics of HIV positive children at ART initiation in public health facilities of Bahirdar city, Northwest Ethiopia, 2021(*N* = 459)

Variables	Categories	Censored n (%)	Initial regimen change n (%)
Age	≤36 months	41(41.41)	58(58.59)
	> 36 months	103(28.61)	257(71.39)
Sex	Male	70(28.57)	175(71.43)
	Female	74(34.58)	140(65.42)
Parental aliveness	One or both parent	138(31.15)	305(68.85)
	Both died	6(37.50)	10(62.50)
Primary care giver	Parent	126(30.07)	293(69.93)
	Non-parent	18(45.00)	22(55.00)
Primary care giver HIV status	Positive	118(29.87)	277(70.13)
	Negative or unknown	26(40.63)	38(59.37)
Primary HIV confirming services	PMTCT	26(40.63)	38(59.37)
	Non-PMTCT	118(29.87)	277(70.13)
Previous ART drugs exposure	Yes	25(43.10)	33(56.90)
	No	119(29.68)	282(70.32)
Catchment residence	Yes	111(30.41)	254(69.59)
	No	33(35.11)	61(64.89)

*Hint* ART-antiretroviral therapy, *HIV* human immunodeficiency virus, *PMTCT* Prevention of mother to child transmission

### Base line clinical, laboratory and treatment related characteristics

Base line clinical, laboratory and treatment related characteristics of the participants were summarized (Table 2).

**Table 2:** Base line characteristics of HIV positive children at ART initiation in public health facilities of Bahir Dar city, Northwest Ethiopia, 2021(*N* = 459)

Variables	Categories	Censored, n (%)	Initial regimen change, n (%)
Opportunistic infection	Yes	73(29.92)	171(70.08)
	No	71(33.02)	144(66.98)
Functional status)	No or mild limitation	121(31.84)	259(68.16)
	Moderate or severe limitation	23(29.11)	56(70.89)
CD4 count	≤200 cells/m^3^	19(35.19)	35(64.81)
	> 200 cells/m^3^	125(30.86|)	280(69.14)
Weight	≤20 kg	88(32.47)	183(67.53)
	> 20 kg	56(29.79)	132(70.21)
Serum hemoglobin	≤11 mg/dl	26(40.63)	38(59.38)
	> 11 mg/dl	118(29.87)	277(70.13)
History tuberculosis	Yes	11(21.15)	41(78.85)
	No	133(32.68)	274(67.32)
ART regimen	TDF based	12(30.00)	28(70.00)
	ABC based	35(60.34)	23(39.66)
	AZT based	81(30.34)	186(69.66)
	D4T based	16(17.02)	78(82.98)
Deterioration of WHO clinical stage	Yes	2(25.00)	6(75.00)
	No	142(31.49)	309(68.51)
Adherence	Good	129(36.54)	224(63.46)
	Poor/fair	15(14.15)	91(85.85)
ART regimens	NVP based	62 (24.41)	192(75.59)
	EFV based	55 (31.61)	119(68.39)
	DTG based	5 (83.33)	1(16.67)
	LPVr based	22(88.00)	3(12.00)
Nutritional status	Severe and moderate under nutrition	55(26.44)	153(73.56)
	Normal and mild under nutrition	89(35.46)	162(64.54)

*Hint* WHO-world health organization, *NVP* Nevirapine, *EFV* Efavirenz, *DTG* Dolutegravir, *LPVr* Lopinavir, *ART* antiretroviral therapy

### Regimen combinations and outcomes

Majority of the ART drug combinations (93.9%) were non nucleotide reverse transcriptase inhibitors based (NNRTI) and the rest 5.23 and 0.87% were protease inhibitor based and integrase strand inhibitor based regimen, respectively. The initial regimens were AZT/3TC/NVP (35.1%), AZT/3TC/EFV (22.7%), D4T/3TC/NVP (16.3%), TDF/3TC/EFV (7.63%), ABC/3TC/LPVr (5.01%), D4T/3TC/EFV (4.14%), TDF/3TC/DTG (0.2%), ABC/3TC/DTG (0.9%) and other regimens (9.12%).

Among the study participants, the ART outcomes during the follow-up time were regimen change (68.63%), transfer out (16.56%), on follow-up (11.98%,), died(1.74%) and lost to follow-up(1.09%).

### Incidence and survivorship status of initial regimen change

**Fig. 2:**
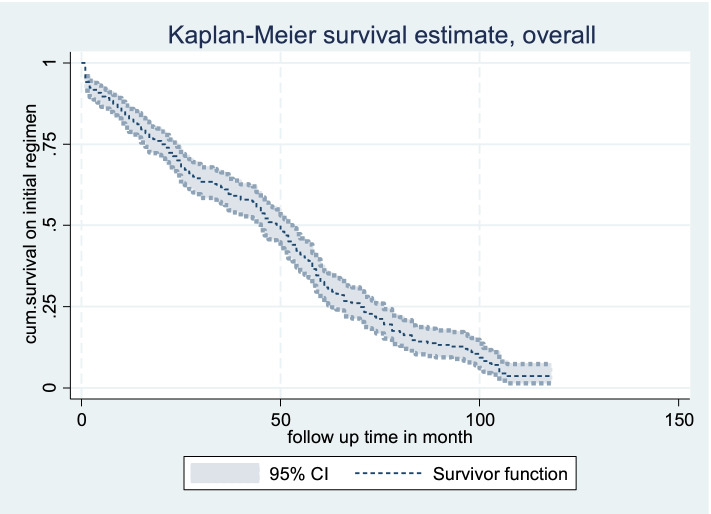
Kaplan Meier curve estimate of overall survival status of children on initial regimen in public health facilities of Bahir Dar city, Northwest Ethiopia,2021

The overall incidence rate of initial regimen change was 1.85, 95% CI (1.66, 2.07) per 100 PMO over a total of 17,010 person-months of follow-up. The overall median survival time was also 49 months, 95%CI (45, 53). The shortest and longest follow-up time was 1 month and 118 months, respectively. The Kaplan- Meier estimation showed that overall estimated survival on initial regimen after starting ART was about 90% at 6 months of follow up. The estimated cumulative survival on initial regimen at 6, 12, 24, 36, 60 months and end of follow-up were 90, 84, 70, 61, 32 and 3%, respectively as showed in Kaplan-Meier survival estimate curve (Fig. 2).

Incidence rate and median survival time of initial regimen change per groups of variables is summarized below (Table 3).

**Table 3:** Incidence rate and median survival time of initial regimen change per groups of variables among children in public health facilities of Bahir Dar city, Northwest Ethiopia, 2021(*N* = 459)

Variables	Categories	IR of IRC per 100 PMO	Median (IQR)
Sex	Male	1.95(1.68, 2.26)	46(37, 52)
	Female	1.75(1.48, 2.06)	52(45, 57)
History tuberculosis	Yes	2.48(1.82, 3.36)	34(17, 52)
	No	1.78(1.59 2.01)	50(45, 54)
Adherence	Good	1.66(1.46,1.90)	57(46, 56)
	Poor/fair	2.57(2.09, 3.15)	37(25, 47)
ART drug regimen	ABC/3TC/EFV	1.89(1.05,3.41)	43(19, 60)
	AZT/3TC/EFV	1.37(1.07, 1.73)	54(50, 64)
	AZT/3TC/NVP	1.79(1.49, 2.14)	57(53, 59)
	TDF/3TC/EFV	1.68(1.12, 2.50)	47(33, 56)
	ABC/3TC/NVP	2.11(1.14, 3.93)	25(18, 46)
	D4T/3TC/NVP	3.39(2.64, 4.36)	22(13,33)
	D4T/3TC/EFV	3.26(2.02, 5.24)	17(13, 28)

*Hint* ABC-abacavir, *ART* antiretroviral therapy, *AZT* zidovudine, *3TC* lamuvidine, *D4T* stavudine, *DTG* Dolutegravir, *EFV* Efavirenz, *IR* Incidence rate, *LPVr* Lopinavir, *NVP* Nevirapine, *TDF* tenofavir disoproxil fumarate, *IRC* initial regimen change

### Predictors of initial ART regimen change

In the multi variable Cox regression analysis, sex, adherence, history of TB and regimen type found to be predictors of initial regimen change (Table 4). In this regard, children who had history of poor or fair adherence of ART were 49% more likely to develop initial regimen change as compared to those who had history of good adherence (AHR = 1.49, 95%CI: (1.16, 1.92)). The hazard of initial regimen change among children who had history of TB were 59% more likely as compared to the counter parts (AHR =1.59, 95%CI: (1.14, 2.23)). In addition, those children initiated ART with NVP regimen at base line were 45% more likely to develop initial regimen change as compared to EFV based regimen (AHR =1.45, 95%CI:(1.15, 1.84)). Moreover, those children on ART who initiated LPVr based regimen were 78% less likely to change their initial regimen as compared to those children who initiate ART with EFV based regimen (AHR = 0.22, 95%CI: (0.07, 0.70)).

**Table 4:** Cox regression analysis of initial ART regimen change predictors among children in public health facilities of Bahir Dar city, Northwest Ethiopia, 2021(*N* = 459)

Variables	Categories	Censored n (%)	IRC (event) n (%)	CHR (95%CI)	AHR (95%CI)	*P*-value
Sex	Male	70(28.57)	175(71.43)	1.14(.92,1.43)	1.28(1.02, 1.60)	.03
	Female	74(34.58)	140(65.42)	1	1	.
Functional status	No limitation	121(31.84)	259(68.16)	1	1	.
	Moderate or severe limitation	23(29.11)	56(70.89)	1.31(.98,1.75)	1.30(.97, 1.74)	.079
History tuberculosis	Yes	11(21.15)	41(78.85)	1.46(1.05, 2.03)	1.59(1.14,2.23)	**.**007
	No	133(32.68)	274(67.32)	1	1	.
WHO clinical stage deterioration	Yes	2(25.00)	6(75.00)	1.78(.79,4.00)	1.69(.74, 3.83)	.210
	No	142(31.49)	309(68.51)	1	1	.
Adherence	Good	129(36.54)	224(63.46)	1	1	.
	Poor/fair	15(14.15)	91(85.85)	1.52(1.2,1.95)	1.49 (1.16, 1.92)	.002
ART drug regimen	NVP based	62 (24.41)	192(75.59)	1.32(1.05,1.66)	1.45(1.15, 1.84)	.002
	EFV based	55 (31.61)	119(68.39)	1	1	.
	DTG based	5 (83.33)	1(16.67)	.99(.14,7.2)	.34(.05, 2.54)	.296
	LPVr based	22(88.00)	3(12.00)	.38(.12, 1.12)	.22(.07, .70)	.011

*Hint* DTG-Dolutegravir, *EFV* Efavirenz, *IRC* initial regimen change, *LPVr* Lopinavire, *NVP* Nevirapine

The adjusted hazard of being male for initial regimen change was 1.28 times more likely as compared to being female (AHR = 1.28, 95%CI: (1.02, 1.60)).

## Discussion

This retrospective follow-up study was aimed to determine the incidence and predictors of initial ART regimen change in Bahir Dar city, Ethiopia. Based on this objective, the overall incidence of initial ART regimen change was 1.85per 100 PMO which were predicted by adherence level, regimen type, tuberculosis and sex of child.

The incidence rate was higher than study done in sub Saharan Africa (0.41per 100 PMO) [16] and Mali (0.28 per 100 PMO [24]. The discrepancy could be due to studies in sub Saharan Africa and Mali were about only switching to second-line ART but this study included both drug substitution and switching to second-line regimen. Furthermore, treatment failure had been diagnosed through the level of CD4 count and WHO clinical stage in the earlier years of HIV care and treatment especially in developing countries but currently, WHO recommends routine viral load test which is important for early diagnosis of treatment failure to meet the 95–95-95 UNAIDS goals [25, 26]. As the finding of this study revealed, the clinicians need to careful for their practice of individual regimen management whether they have been adhered to standards and evidence-based practice along with the national guideline while changing the regimens or not.

The finding of this study showed that the hazard of initial regimen change among children who initiate NVP based regimen was higher than those children who initiated EFV based ART regimen. The finding was supported by large sample study in France [27] and multicenter study in Europe and Thailand [14]. This risk difference might be due to NVP toxicity. NVP toxicity is associated with immune competency results in NVP based regimen to have higher regimen change rate [28]. This could be after WHO recommendations of ART initiation regardless of CD4 count in children [29]. In addition, the finding was deferred from the study done in Malaysia which stated that NNRTI based categories of regimen had no difference for regimen change [3]. This could be due to sample size and analysis model used by this study. The risk of regimen change difference between NVP and EFV based regimens could be due to the adverse effect of EFV more frequently occur within months and self-resolved without regimen change within months. Additionally, EFV has high half life than NVP which result in low frequency of daily dose and good adherence [30]. Besides, the hazard difference between these drugs could be due to the CD4 dependent serious side effect of NVP and its self inducing characteristics of its metabolism [30]. The other hazard difference between the two groups might be due to the high barrier resistance of EFV and its viral suppressing effect over NVP [31]. On the other hand, those children who started LPVr based regimen had lower hazard of initial regimen change than EFV based regimens which is consistent with study done in Thailand [14]. This could partly mirror the reluctance of clinicians to switch children failing LPVr based regimen due to the difficulty in deciding what to switch to, and recommendations to first address adherence issues due to the high resistance barrier and guideline restriction for children aged less than 3 years [26, 32]. This implies that LPVr based regimen is preferable than EFV and NVP based regimens to advance the potential durability of the initial regimen which supports the current WHO recommendation of ARV drug preference lists.

In this study, the hazard of initial regimen change among children who had poor adherence was higher than those children who had good adherence. The finding was similar with the study done in Malaysia [3]. The hazard difference between the two groups could be due to immature disclosure especially in old children. In addition, palatability and preparation of the drugs might be lead to poor adherence in this age group. Similarly, frequency of dosing, drug side effects, child’s age and developmental stage, psychosocial, behavioral, and socio-demographic characteristics of children and caregivers might be associated with poor adherence [33]. Furthermore, infants and children are dependent on others for medication administration. As a result adult caregivers may face barriers that contribute to poor adherence in children including forgetting doses, changes in routine, being too busy, and child refusal [34]. This finding showed that despite the current strategies to boost ART adherence practice of the child, adherence is still challenging in clinical practice of HIV care and treatment program.

This study finding also showed that hazard of initial ART regimen change was higher for being male than being female. The finding was comparable with an international cohort collaboration regimen change study reported that male had higher rate of switching to second line regimen over female [35] and the Pediatric International Epidemiology Databases to Evaluate AIDS (IeDEA) study member countries (Asia-pacific and African countries) [36]. The gender discrepancy might be due to psychosocial transition difference. In addition, sex discrepancies in treatment outcomes are thought to result from differences in both biologic and behavioral factors including antiretroviral metabolism, antiretroviral adherence and retention in care. A related study on sex difference in pharmacologic effect of antiretroviral therapy among adult reported that sex-related variations in the expression and activities of drug transporter genes, proteins, and enzymes involved in phases of the drugs biotransformation [37]. The difference in maintaining initial ART regimen between male and female child implies the requirement of further close supervision for male child by clinicians and other responsible bodies.

Moreover, the finding of this study revealed that the hazard of initial regimen change among children who has history of tuberculosis was higher than those children who hadn’t history of tuberculosis. The finding was consistent with studies conducted in Ghana [11] and Uganda [38]. The hazard difference between the two groups could be due to the occurrences of adverse effect of ART drugs together with the extra dose frequencies of both drugs (ART and anti-TB) which lead to poor adherence [39, 40]. In addition, TB-HIV co infection may impair or delay immune recovery after ART initiations. Furthermore, TB is the most frequent life-threatening opportunistic infection and the leading cause of death among HIV positive children [5]. TB speeds up the viral replication in HIV infected individuals which in turn leads to treatment failure due to ineffective antiretroviral drugs [26]. In addition, the initial ART regimen could be changed due to drug-drug interaction in children who developed tuberculosis while they were on ART [41].

Since the data were collected from secondary source, some important predictors such as family’s wealth index, viral load at baseline and care giver educational level were missed. Additionally, it also missed some important variables like laboratory diagnostic tests (blood chemistry, organ function test) and some socioeconomic factors. Furthermore, it might be prone for selection bias. Another limitation of this study could be over estimation of incidence of initial ART regimen change due to inclusion of all ART regimen changes following national guideline changes.

## Conclusions

In a conclusion, the incidence of initial regimen change was high as compared to the previous studies. The possibility of initial regimen change is predicted for HIV positive children who had initiated ART with history of tuberculosis, NVP based regimen, being male by sex and poor adherence. LPVr based regimen is preferable than EFV and NVP based regimens to advance the potential durability of the initial regimen which supports the current WHO recommendation of ARV drug preference lists. Therefore, strengthening the health care providers’ adherence counseling capability, strengthening tuberculosis screening and prevention strategies and care of initial regimen type choice needs attention in the HIV/AIDS care and treatment programs. Moreover, further prospective study is recommended to be conducted by including family’s wealth index, viral load at baseline, care giver educational level, hepatic function test and renal function test.

## Data Availability

The datasets used and/or analyzed during the current study are available from the corresponding author (MAB) on reasonable request.

## Abbreviations

ABC: Abacavir
ADR: Adverse Drug Reaction
AIDS: Acquired Immunodeficiency Syndrome
ART: Anti Retroviral Therapy
ARTRC: Anti Retroviral Therapy Regimen Change
AZT: Azithymidine/zidovudine
CD4: Cluster of differentiation 4 T helper cell
CI: Confidence Interval
D4T: Stavudine
EFV: Efavirenz
HAART: Highly Active Anti Retroviral Therapy
HIV: Human Immunodeficiency Virus
LPV/r: Ritonavir boosted Lopinavir
NVP: Nevirapine
PMO: Person month observation
RC: Regimen Change
TB: Tuberculosis
TDF: Tenofovir Disoproxil Fumarate
UNAIDS: Joint United Nations Program on HIV/AIDS
WHO: World Health Organization
3TC: Lamivudine
